# Toxic Effects Produced by Anatoxin-a under Laboratory Conditions: A Review

**DOI:** 10.3390/toxins14120861

**Published:** 2022-12-08

**Authors:** Cristina Plata-Calzado, Ana I. Prieto, Ana M. Cameán, Angeles Jos

**Affiliations:** Area of Toxicology, Faculty of Pharmacy, Universidad de Sevilla, Profesor García González 2, 41012 Seville, Spain

**Keywords:** Anatoxin-a, *in vitro*, *in vivo*, toxicity, laboratory studies

## Abstract

The presence of cyanotoxins and its bioaccumulation in the food chain is an increasingly common problem worldwide. Despite the toxic effects produced by Anatoxin-a (ATX-a), this neurotoxin has been less studied compared to microcystins (MCs) and cylindrospermopsin (CYN). Studies conducted under laboratory conditions are of particular interest because these provide information which are directly related to the effects produced by the toxin. Currently, the World Health Organization (WHO) considers the ATX-a toxicological database inadequate to support the publication of a formal guideline reference value. Therefore, the aim of the present work is to compile all of the *in vitro* and *in vivo* toxicological studies performed so far and to identify potential data gaps. Results show that the number of reports is increasing in recent years. However, more *in vitro* studies are needed, mainly in standardized neuronal cell lines. Regarding *in vivo* studies, very few of them reflect conditions occurring in nature and further studies with longer periods of oral exposure would be of interest. Moreover, additional toxicological aspects of great interest such as mutagenicity, genotoxicity, immunotoxicity and alteration of hormonal balance need to be studied in depth.

## 1. Introduction

The presence of cyanotoxins and their bioaccumulation in the food chain is an increasingly common problem worldwide [1]. Anatoxin-a (ATX-a) is a cyanotoxin synthesized by various members of the genera *Anabaena* [2], *Aphanizomenon* [3], *Cylindrospermum* [4], *Microcystis* [5], *Oscillatoria* [4], *Planktothrix* [6] and *Raphidiopsis* [7,8]. The occurrence of ATX-a has been reported in USA [9], Africa [10,11], Asia [5,8,12] and Europe [6,13,14,15]. Although ATX-a-producing species have been found in freshwater sources distributed throughout the world, this cyanotoxin has been less studied compared to other cyanotoxins such as MCs and CYN [16].

**Figure 1:**
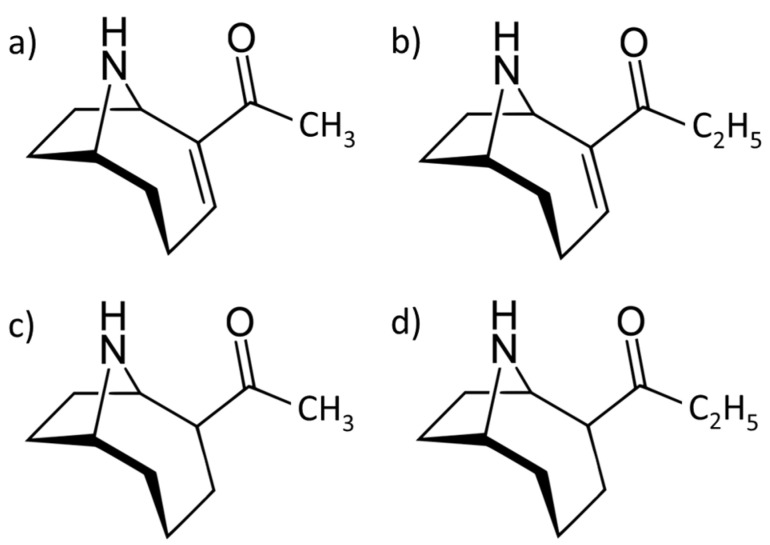
Structures of (**a**) ATX-a and analogues/derivatives: (**b**) homoanatoxin-a, (**c**) dihydroanatoxin-a, (**d**) dihydrohomoanatoxin-a.

Structurally, it is a relatively small molecule with two enantiomeric forms (Figure 1) and an average molecular weight of 165.237 g/mol, chemical formula C_10_H_15_NO, a pKa of 9.36 for the (+)ATX-a enantiomer and a K_ow_ value of 0.8, so it is protonated at physiological pH and highly soluble in water. ATX-a is unstable in natural conditions and mainly at high temperatures (100 °C) and basic pH (9.5), degrading into its 2,3-epoxy-, 4-hydroxy- and 4-oxo-derivatives. Moreover, ATX-a has several analogues, such as homoanatoxin-a, dihydroanatoxin-a and dihydrohomoanatoxin-a [17,18]. In relation to mechanisms of action, ATX-a is a potent inhibitor of the enzyme acetylcholinesterase (AChE) by binding to neuronal receptors of acetylcholine (nAChR). It causes membrane depolarization by opening this receptor channel, leading to a blockade of neuromuscular transmission. Moreover, acetylcholine is released, producing continuous muscle stimulation [7,19].

There are many ways in which humans may be exposed to cyanotoxins, and the consumption of contaminated water is the main route of exposure for the general population. The presence of ATX-a in food and dietary supplements is also generating great interest. However, although to a lesser extent, recreational use of lakes and rivers may involve an important route of exposure in certain environments. Regarding the reported presence of ATX-a, of all water bodies, the highest frequency of this toxin has been recorded in reservoirs, which raises concern [20].

Currently, there are still very little scientific works on the bioaccumulation of ATX-a. Despite the evidence of accumulation of other cyanotoxins, the hypothesis of this toxin bioaccumulation is recent and has been underexplored [17]. However, the bioconcentration capacity of ATX-a has been confirmed by Osswald et al. [21] in juvenile fish. They exposed fish for 96 h to three concentrations of ATX-a (132, 264 and 524 µg/L), showing a bioconcentration factor ranging from 30 to 47 based on fresh weight. Moreover, bioaccumulation of ATX-a has been demonstrated in three species of common freshwater fish under natural conditions [22]. These authors detected ATX-a accumulation (up to 30 ng/g FW) in fish muscles, suggesting the probability of its transfer to the food chain. In addition, this cyanotoxin has also been detected in other aquatic organisms such as benthic *Chironomus* and aquatic plants [23,24]. On the other hand, algae-based supplements are becoming increasingly popular due to their beneficial health effects. In this respect, the presence of ATX-a has been shown in 7.7% of the samples analyzed by gas chromatography–mass spectrometry (GC-MS) in a concentration range of 2.50–33 µg/g [25]. As mentioned above, only a few studies focused on bioaccumulation and contamination, which are essential for risk assessment, have been performed.

Numerous cases of human and animal intoxication due to accidental exposure to ATX-a have been described. Thus, some of the symptoms derived from poisoning by this toxin are urinary incontinence, ataxia, asthenia, lacrimation, salivation, blurred vision, dizziness, muscle cramps, headache, paresthesia, respiratory failure, convulsions, and cerebral hypoxia [26,27]. Moreover, the data related to the toxicokinetics of ATX-a are scarce so far. However, the acute oral toxicity studies in animals suggest that this neurotoxin is rapidly absorbed from the gut due to the symptoms of neurotoxicity occurring within minutes of exposure [28,29]. There are also no studies focused on ATX-a distribution, metabolism and excretion. In relation to the distribution process, ATX-a can be distributed rapidly to the central and peripheral nervous system, and is able to cross the blood–brain barrier. Some authors have shown increased ethoxyresorufin-O-deethylase (EROD) and Glutathione S-transferase (GST) enzyme activities, suggesting that ATX-a could undergo phase I and II metabolism [30]. In addition, there are indications that ATX-a can be partially eliminated in an unchanged form, but studies are necessary in this regard [16].

To date, the involvement of ATX-a in numerous animal poisonings worldwide has been extensively reviewed [17]. Specifically, major episodes of ATX-a poisoning have been identified predominantly in dogs [14,31,32,33,34,35,36,37,38], but they have also been described in other animal species such as cows, flamingos, ducks or carps [17]. However, field studies usually lack accurate qualitative and quantitative information regarding the cyanotoxins involved in intoxications and the results can be biased by cofactors including other environmental contaminants or previous diseases of the animals. In fact, the variety of co-occurring symptoms may suggest that several cyanotoxins could be involved in the intoxication process [17]. Moreover, the irrefutable imputation of a poisoning to a single toxin often lacks evidence because the same cyanobacterial species can produce different kind of toxins and the coexistence of cyanotoxins is frequent in nature [39,40,41]. Likewise, other reviews mainly consider other aspects such as the chemistry of ATX-a and its congeners, the factors influencing its production, its bioaccumulation in different matrices, biosynthesis, degradation, etc., but do not go into toxicity studies under laboratory conditions [9,17,42]. Therefore, results derived from studies conducted under laboratory conditions are of particular interest. These studies provide data that are directly related to the effects produced by the toxin under certain exposure conditions (concentration, exposure time, via/route, etc.). To our knowledge, this is the first review focusing on *in vitro* and *in vivo* studies performed thus far on ATX-a. Despite the fact that WHO has recently provided provisional reference values of 30 μg/L ATX-a for acute or short-term exposure via drinking-water and 60 μg/L ATX-a for recreational water exposure, this organization recognizes that the current ATX-a toxicological database is not adequate to support the publication of a formal guideline reference value [16]. This highlights the need for further studies focusing on the potential toxicity of ATX-a under controlled conditions.

The aim of the present work is to compile the *in vitro* and *in vivo* studies performed under laboratory conditions so far. This is essential to being able to unify criteria and to lay the toxicological foundations as a starting point for new studies focused on the research of the toxic effects of ATX-a.

## 2. *In Vitro* Toxicity Studies

In general, there are few *in vitro* studies focused on investigating ATX-a toxicity (Table 1).

**Figure 2:**
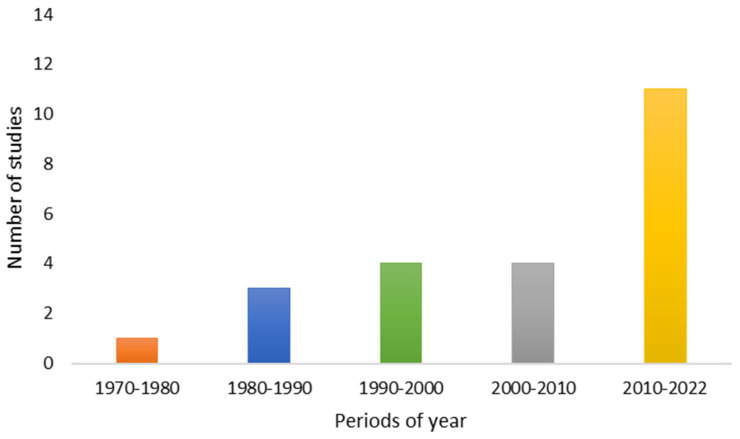
Number of *in vitro* studies published in relation to years of publication.

**Figure 3:**
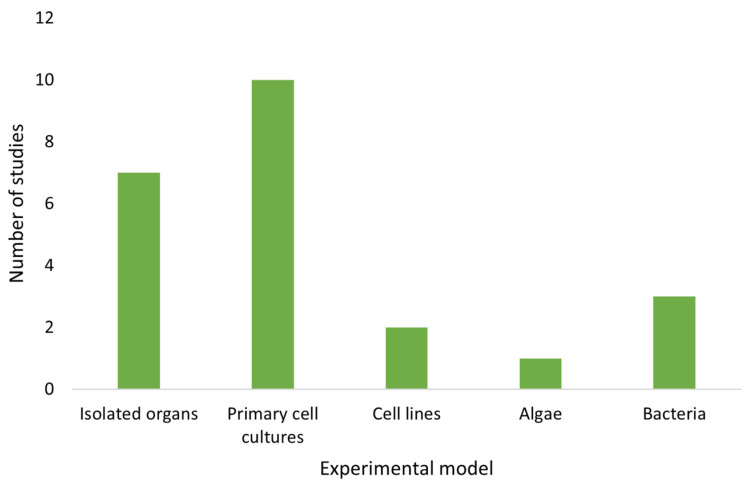
Number of *in vitro* studies published in relation to the experimental models used.

However, the use of these assays for ATX-a research has increased significantly from the 2010s to the present, indicating that it is a scarcely studied topic but of growing interest (Figure 2). The first *in vitro* studies on ATX-a were mostly performed on isolated organ models (Figure 3).

The main objective of these studies was to demonstrate the mechanism of action and the potency of this toxin by comparing it with other substances such as acetylcholine, carbachol [43] or decamethonium [44]. These authors showed that ATX-a has the greatest ganglionic stimulatory effects on smooth muscles in guinea pig ileums [43] and a low affinity for muscarinic acetylcholine receptors in rat brains [44]. Moreover, Swanson et al. [45] demonstrated that ATX-a presents a high affinity for the nicotinic acetylcholine receptor, however this affinity was isomer-dependent, as (+)ATX-a is 160 times more potent than (−)ATX-a in inhibiting acetylcholine binding in frog muscle [46]. Similarly, Thomas et al. [47] described (+)ATX-a as the most effective nicotinic agonist, estimating that it was between 3 and 50 times more potent than (−)-nicotine and 20 times more potent than acetylcholine in fetal rat hippocampal neurons. Later, Fawell et al. [50] observed a different agonist potency with respect to nicotine depending on the organ studied. Thus, the largest differences were found in the rat phrenic nerve diaphragm where ATX-a was a 136-fold more potent agonist than nicotine, followed by the chicken biventer neck and guinea pig ileum (24- and 7-fold, respectively).

Moreover, other authors confirmed that this toxin is involved in the release of excitatory neurotransmitters such as noradrenaline and adrenaline in bovine adrenal chromaffin cells and in slices of the hippocampus, thalamus and cortex [48,51]. In addition, ATX-a also produces a Ca^2+^-dependent release of monoamides such as dopamine in rat synaptosomes [49].

An advance in the knowledge of the mechanism of toxic action of ATX-a indicated that the apoptosis produced by (+)ATX-a (pure and from an extract of *Anabena flos-aquae*) in rat thymocytes and Vero cell lines was mediated by activation of the caspase chain and the generation of reactive oxygen species (ROS) in a concentration- and time-dependent manner [52]. Similarly, other authors have more recently demonstrated the production of oxidative stress by alteration of antioxidant parameters such as malondialdehyde (MDA), ROS, superoxide dismutase (SOD), catalase (CAT), glutathione reductase (GR), glutathione peroxidase (GPx) and glutathione (GSH) in goldfish lymphocytes [63]. Moreover, these authors also confirmed cellular cytotoxicity evidenced by apoptosis and DNA fragmentation [63]. In this regard, Teneva et al. [53] also demonstrated cytotoxic effects produced by ATX-a on T and B lymphocytes isolated from mice at similar concentrations and periods of exposure (4 h). However, longer exposure times were necessary to observe cell apoptosis in common carp lymphocytes (24 h) at similar toxin concentrations [55]. N2a neuroblastoma cells showed higher sensitivity to ATX-a-induced cytotoxicity (alone or in mixture with MC-LR or CYN) than BV-2 cells and RAW264.7 cells in a concentration range of 0.001–10 µM. However, the LD_50_ was only reached with the cyanotoxin mixture at different exposure periods (24, 48 and 72 h) [60]. More recently, a significant decrease in cell proliferation and cytotoxicity produced by ATX-a in human keratinocytes has been demonstrated by Adamski et al. [64]. In general, significant cytotoxic effects produced by ATX-a are observed in different cell lines; however, despite ATX-a being classified as a neurotoxin, only one of these cell lines is of neuronal origin.

Conversely, ATX-a has been shown to alter cytokine production in common carp leukocytes differently depending on the origin of the toxin (pure or from a cyanobacterial extract) [57]. Neuronal cells (N2a cells) exposed to ATX-a have shown a higher ability to produce tumor necrosis factor (TNF-α) than others such as RAW264.7 and BV-2 cells when these cell lines are compared [60]. The immunotoxicity of ATX-a has been recently challenged. However, only *in vitro* data are available to estimate the immunotoxicity of ATX-a. For this, more studies are needed in this regard, as we cannot rule out some detrimental consequences of ATX-a over the immune system [17].

Regarding the evaluation of the genotoxic potential of this toxin, studies performed have been scarce and incomplete. Although ATX-a is classified as a neurotoxin, it could also show genotoxic potential as it has been demonstrated for other cyanotoxins such as MC-LR and CYN [65]. In this sense, the European Food Safety Authority (EFSA) has recommended, as a first step, the performance of a basic battery of *in vitro* tests including the Ames Test and the micronucleus test (MN) [66]. Thus, until now, none of the studies carried out with ATX-a comply with the full performance of this proposed battery of tests, and the reported results are contradictory. For example, Abramsson-Zetterberg et al. [67] performed the MN on human lymphocytes in the absence of S9 for a cyanotoxin extract in a concentration range of 0.25–10 mg/mL and observed no genotoxic effects. However, these authors did not confirm the presence of ATX-a in these extracts. Subsequently, different tests with *Salmonella typhimurium* were carried out. Thus, Sieroslawska and Rymuszka [54] observed genotoxicity after ATX-a exposure in strain TA1535 only in the absence of S9 using the UmuC Easy CS assay [59]. Nevertheless, pure ATX-a showed no mutagenicity in a concentration range of 0.312–10 µg/mL in strains TA98, TA100, TA1535, TA1537 and *E. coli*, while an extract of this toxin showed mutagenicity in strains TA98 and TA100 [58]. In contrast, no genotoxic effects were observed in the comet assay on carp leukocytes exposed to 0.5 µg/mL ATX-a. Due to the importance of the consequences of genotoxicity of the substances on consumers, studies following EFSA recommendations on the genotoxic aspects of ATX-a alone and in mixture with other cyanotoxins are needed.

In other experimental models such as yeasts (*Saccharomyces cerevisae*)*,* ATX-a has also been shown to be an estrogenic agonist by modulating 17β-estradiol-induced estrogenic activity in the YES assay [61]. However, this is the only study on the subject and it is an interesting field to explore. Conversely, in algae, ATX-a produces different effects on cell density depending on the species. Exposure to 25 µg/L of ATX-a (alone or in mixture with MC-LR) decreases the cell density of *Microcystis* sp., increases that of *Selenastrum capricornutum* and does not change that of *Anabaena* [62]. In general, it has been shown that the toxic effects produced by ATX-a are more intense when this toxin is combined with MC-LR and/or CYN [60,62]. This is important to note because in nature ATX-a is not usually found isolated but in combination with other cyanotoxins.

Finally, only one study has used different species of blue-green algae as experimental models [62].

## 3. *In Vivo* Experimental Studies

**Figure 4:**
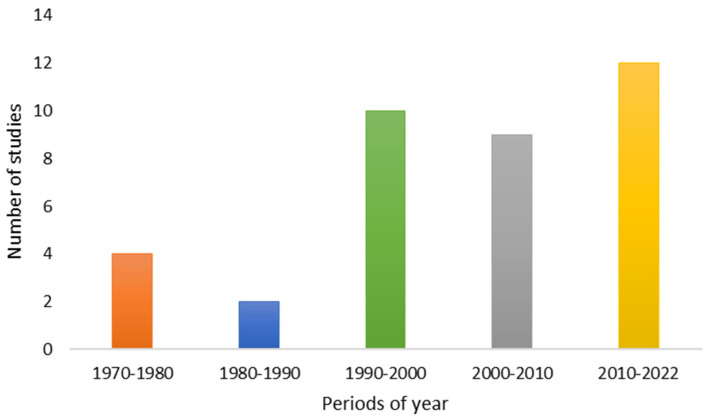
Number of *in vivo* studies published in relation to years of publication.

Based on the number of published *in vivo* studies on ATX-a, it is observed that there has been an increase in studies using *in vivo* models since the 1990s (Figure 4). Although these studies constitute a more advanced stage in the investigation of toxicological effects, it is noted that the increase in the use of *in vivo* models was earlier than that observed in the use of *in vitro* models (since 2010–2022) (see Figure 2 and Figure 4).

**Figure 5:**
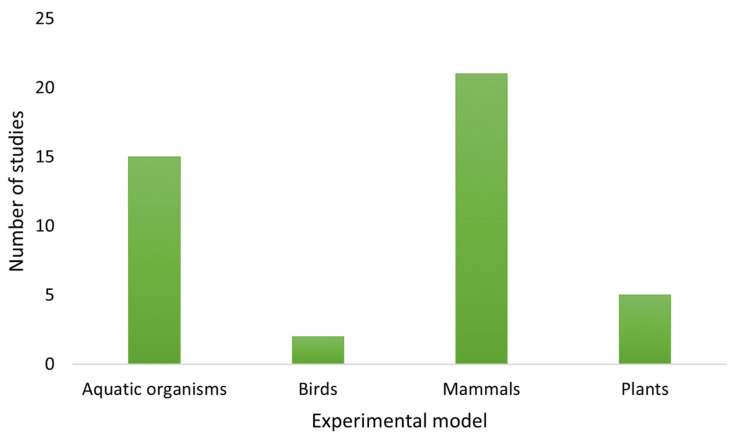
Number of *in vivo* studies published in relation to the experimental models used.

Moreover, several *in vivo* laboratory studies have been carried out in a range of animal models such as aquatic animals, birds, mammals and plants, in order to elucidate the toxic effects produced by ATX-a (see Table 2 and Figure 5). Nevertheless, more than half of the studies have been conducted in rodents versus other animal models such as fish, birds or plants. Carmichael and Biggs [68] were pioneers in the study of ATX-a toxicity, reporting a different sensitivity depending on animal model, with goldfish as the most sensitive species, followed by duck, calf, pheasant, rat and mouse.

**Table 2:** *In vivo* laboratory toxicity studies carried out with ATX-a in different experimental models.

Experimental Model	Experimental Conditions	Assays Performed	Main Results	References
Aquatic Animals				
Goldfish (and other species, see birds and mammmals)	Oral or i.p. doses of *Anabaena flos-aquae* NRC-44-1 or immersion in an aqueous medium containing 6 µg/mL toxin extract for 8 h	Clinical observations	Death was produced by respiratory arrest after 12–14 min when administration was orally or i.p. No adverse effects were observed when fish were placed in an aqueous medium containing the toxin.	[69]
Goldfish (and other species, see birds and mammmals)	I.p. injection or oral doses of *Anabaena flos-aquae* NRC-44-1 containing ATX-a	Determination of LD_90_	When administration was oral, goldfish were the most sensitive species to ATX-a (LD_90_ = 120 mg/kg). The i.p. LD_90_ was half that of the oral dose (LD_90_ = 60 mg/kg).	[68]
Brine shrimp (*Artemia salina)*	25 or 50 µg/mL of pure ATX-a, 20 µg ATX-a per mg of nontoxic *Anabaena* or *Anabaena* strains containing ATX-a	Toxicity determination by *Artemia salina* biotest	Concentration up to 50 µg/mL of pure ATX-a were not toxic to *Artemia* larvae, although when ATX-a was mixed with nontoxic *Anabaena,* an increase in the death percentage of the larvae was observed. This result may indicate that ATX-a was not the responsible compound of that toxicity. Abnormal movements were observed with *Anabaena* strains containing ATX-a.	[70]
Brine shrimp (*Artemia salina*)	0–100 mg/L *Anabaena* strains containing ATX-a or cyanobacterial bloom	Toxicity determination by *Artemia salina* biotest	ATX-a only produced abnormal swimming in the *A. salina* bioassay, whereas *Anabaena* strains containing ATX-a caused mortality (LC_50_ = 2–14 mg/L).	[71]
Zebrafish embryos	Concentrations of 40, 200 or 400 µg/L ATX-a and exposure to crude extracts of cyanobacteria	Heart rate measurement and malformation observation	The highest concentration produced temporary alterations in heart rates. No chronic effects were observed. No effects were observed with the crude extract in which ATX-a was detected.	[72]
Embryos of toads *(Bufo arenarum)*	Amphibian stage 18 embryos were exposed to 0.03, 0.3, 3.0 or 30 mg/L ATX-a for 10 days and stage 25 embryos were exposed to 30 mg/L ATX-a for 10 days.	Embryo-larval toxicity test (AMPHITOX)	Toad embryos shown a concentration-dependent transient narcosis, oedema and loss of equilibrium as adverse effects, and a mortality of 100% at the highest concentration in both groups 6–13 days post-exposure.	[73]
*Cyprinus carpio*	10^5^ cel/mL or 10^7^ cel/mL of *Anabaena* containing ATX-a for 4 days	Study of behavioral and bioaccumulation of toxin by HPLC	Treated carps showed behavior alterations. The highest cyanobacteria concentration caused the death of all fish, whereas with the small one, no deaths were observed. The highest level of toxin detected in the whole fish was 0.768 µg/g of carp weight.	[74]
Fertilized eggs from *Cyprinus carpio*	Fertilized eggs were incubated over 4 days with cyanobacterial cell extract of *Anabaena* sp. (6.6 × 10^5^–8.3 × 10^4^ cell/L that correspond to 83.3–666 µg/L ATX-a) or pure ATX-a (80–640 µg/L)	Registration of mortality analysis of hatching rate and skeletal malformations at 4, 9 and 24 h, and every 24 h for 8 days after the first exposure	Pure toxin only produced a decrease in larval length at the highest concentration. However, concentration-dependent adverse effects were observed with the cyanobacterial extract, producing 100% mortality at the highest concentration.	[75]
Common carp	25 µg/L of ATX-a for 5 days by inmersion	Cytotoxicity by bioluminescent assay and proliferation by DNA fragmentation	Decreased ATP levels were not observed. A reduction in GSH levels and proliferation of T and B lymphocytes in pronephros and blood was produced.	[56]
Rainbow trout (*Oncorhynchus mykiss)*	Range-finding bioassay: Single dose of 0.005–5 µg/g ATX-a by i.p. injection Main test: 0.08–0.31 ATX-a by i.p. injection	Determination of LD_50_ Measurement of enzymatic biomarkers in muscle or liver	Survival after exposure to the lowest doses of the toxin. Death at 30 and 17 min after treatment with 0.5 and 5 µg/g, respectively. The LD_50_ determined was 0.36 µg/g. An increase in AChE and LDH activities in muscle and GST and EROD activities in liver were observed. The rise of these activities in the liver indicated the involvement of phase I and II biotransformation in ATX-a detoxification.	[30]
Zebrafish	Dose of 0.8 µg/g b.w. (±)ATX-a by i.p. injection	Study of behavior and comparison of proteome in brain and muscle between gender by 2DE analysis and mass spectrometry	Fish showed behavior alterations. Males showed more increase in the abundance of proteins than females. Also, differences in protein expression were observed between gender. Proteins that were altered play functions in stress response, detoxification, energy production or cell structure maintenance.	[76]
*Brachionus calyciflorus* and *Daphnia pulex*	0.42, 0.83 or 1.66 mg/L ATX-a for 24 h or cyanobacterial extracts containing ATX-a	Percentage of survivorship in acute toxicity bioassays	Pure ATX-a reduced the survivorship of *D. pulex* to 33% at 1.66 mg/L, whereas in *B. calyciflorus* did not produce effects. Cyanobacterial extracts containing mixtures of different cyanotoxins and other cyanobacterial metabolites were more toxic than pure toxins at lower concentrations.	[77]
*Daphnia magna*	Concentrations ranged from 0.5 to 50 µg/mL ATX-a for 24 h	Swimming response Measurement of oxygen consumption, heart rate and thoracic limb activity	Changes in swimming behavior were noted after treatment. A reduction in a concentration- and time-dependent manner of heart rate, oxygen consumption and thoracic limb activity was observed.	[78]
Female medaka fish (*Oryzias latipes*)	Single dose of 0.2–20 µg ATX-a by gavage	Behavioral study for 30 min Bioaccumulation of toxin in gut, liver and muscle by UHPLC Analysis of liver metabolomes by LC–MS/MS Determination of LD_50_ and NOAEL	The higher dose without effects was 6.67 µg/g and the oral LD_50_ and LD_100_ were 11.5 µg/g and 20 µg/g, respectively. Moreover, fish showed effects such as abnormal swimming and musculature rigidity among others. The content of the toxin decreased rapidly in tissues: after 12 h, ATX-a could not be detected in the liver, or after 3 days in the gut and muscles. Analysis of metabolome suggested a complete recovery 24 h after treatment with a NOAEL dose of toxin.	[79]
*Daphnia magna* clones and newborns from treated *D. magna* clones	Exposure to 100% *Tychonema bourrelyi* containing ATX-a or 50% *T. bourrelyi* + 50% *Scenedemus obliquus* for 4 days by diet	Measurement of juvenile somatic growth rates Quantification of NAR gene expression by qPCR	Treatment with 100% *T. bourrelyi* decreased the somatic growth rate and increased NAR gene expression. In contrast, with 50% *T. bourrelyi*, only a clone showed an increase in NAR expression without changes in growth rate. Moreover, this exposure to mothers affected to their offspring, showing a higher growth rate.	[80]
Birds				
Mallard ducks (and other species, see fish and mammmals)	Oral or i.p. doses of lyophilized *Anabaena flos-aquae* NRC-44-1	Clinical observations	Animals showed opisthotonus and muscular rigidity.	[69]
Chick, mallard duck and ring-necked pheasant (and other species, see fish and mammmals)	I.p. injection or oral doses of *Anabaena flos-aquae* NRC-44-1 containing ATX-a	Determination of LD_90_	When administration was oral, ducks were the most sensitive bird to ATX-a (LD_90_ = 350 mg/kg), followed by pheasants (LD_90_ = 850 mg/kg). Intraperitoneally, pheasants needed 2 times more dose (LD_90_ = 120 mg/kg) than ducks and chicks.	[68]
Mammals				
Calves, rats and mice (and other species, see fish and birds)	Oral or i.p. doses of *Anabaena flos-aquae* NRC-44-1	Clinical observations and determination of MLD	Death was produced by respiratory arrest because of neuromuscular depolarizing activity. Oral MLD of calves was estimated to be 6–8 times higher than that of the mouse i.p. MLD/kg. The time to produce the death was 4–5 min for mice, 7 min for calves and 14–16 min for rats.	[69]
Mouse and rat (and other species, see fish and birds)	I.p. injection or oral doses of *Anabaena flos-aquae* NRC-44-1 containing ATX-a	Determination of LD_90_	The oral LD_90_ for mice and rats were 1800 and 1500 mg/kg, respectively. The i.p. LD_90_ was equal in both species used (LD_90_ = 60 mg/kg).	[68]
Male mice	Oral or i.p. doses of *Anabaena flos-aquae* NRC-44-1 containing ATX-a	Clinical observations and Determination of LD_min_	Animals showed convulsions and tremors. The LD_min_ obtained were 80 mg/kg i.p. and 800 mg/kg orally.	[81]
Calves	Administration of one or sequential doses of *Anabaena flos-aquae* NRC-44-1 by stomach tube	Analysis of blood samples and clinical observations	Loss of muscle coordination and muscle fasciculations were produced. Oral MLD was estimated in 420 mg/kg.	[82]
Female Sprague Dawley rats and pregnant Golden hamsters (*Cricetus auratus)*	Rats were exposed orally to 0.51 or 5.1 µg/mL ATX-a in drinking water for 7 weeks, or to 0.016 mg ATX-a daily i.p. doses for 21 days Hamsters received three i.p. doses of ATX-a at 0.125 or 0.2 mg/kg bw on gestation days 8–11 or 12–14	Gross and microscopic analysis and measurement of enzymatic activities of AP, GPT, GGTP, CE	No adverse effects were seen in rats. Treatment of pregnant hamsters did not cause any malformations but caused stunting at all doses and periods compared with controls in 10–20% of fetuses. No maternal toxicity was observed.	[83]
NMRI-strain female mice	i.p. injections of 2.5–5 mg cyanobacteria blooms containing ATX-a	Determination of toxicity by mouse bioassay	The toxicity was different depending on the bloom sample. *Anabaena* species were present in all neurotoxic samples except one, in which *Oscillatoria* was the dominant species. The MLD obtained ranged from 50 to 500 mg/kg.	[4]
Male Sprague-Dawley rats	Intracerebroventricular or i.v. injections doses of 10, 30, 100 or 300 µg/kg ATX-a	Measurement of cardiac output by thermodilution technique Measurement of organ blood flow by Doppler technique Determination of catecholamines levels by UHPLC	The higher doses of toxin administered i.v. and intracerebroventricular produced a transient increase in cardiac output and vasoconstriction in the renal and mesenteric blood vessels. In addition, plasma epinephrine levels were increased two-fold with the dose of 100 µg/kg ATX-a. These effects were attenuated after chlorisondamine administration, a ganglion blocker.	[84]
Male Balb C mice and male Sprague Dawley rats	Mice were treated i.p. injection of 0.4–0.7 mL (+)ATX-a or (±)ATX-a; i.p. injections 1–73 mg/kg of (−)ATX-a Rats received 50–800 µg/kg of (+)ATX-a by i.v. injection	Behavioral study and measurement of ECAP	The LD_50_ for (+)ATX-a and (±)ATX-a were 386 µg/kg and 913 µg/kg, respectively. No deaths were observed with (−)ATX-a. The ED_50_ for depression of the ECAP was 47 mg/kg and the effects were dose-dependent.	[85]
Male Swiss Webster ND-4 mice	Daily single dose for 4 days or four doses in a day of pure (+)ATX-a or ATX-a derived from two different cyanobacterial extracts administered orally or i.p.	Determination of LD_50_	More levels of toxin were necessary to produce death by oral route. The LD_50_ obtained was similar for all treatments when the administration was i.p. (0.23–0.28 mg/kg ATX-a). However, extract from *Anabaena flos-aquae* NCR-44-1 was 2-fold more potent (6.3–7.1 mg/kg) by oral route than pure toxin (15.4–17 mg/kg).	[28]
Male Sprague Dawley rats	Single i.v. dose ranging from 1 to 500 µg/kg of (+)ATX-a or (±)ATX-a	Measurement of blood pressure, heart rate, blood gases, pH and mortality	Lower doses of (+)ATX-a were necessary to produce the adverse effects. Nevertheless, both produced an increase in blood pressure and a decrease in heart rate, dose-dependent. In addition, hypoxemia, hypercapnia and acidosis were observed. LD_50_ for (+)ATX-a was ≈85 µg/kg and for (±)ATX-a was ≈400 µg/kg.	[86]
Male hooded rats	Subcutaneous injections of 10–200 µg/kg (+)ATX-a	Assessment of locomotor activity for 30 or 60 min	Reduction in locomotor activity either in nicotine-tolerant and non-tolerant rats.	[87]
Mouse	ATX-a was administered by gastric intubation, inhalation or i.p. injection ATX-a + MC-LR by intranasal route	Determination of LD_50_	I.p. injection was the most sensitive administration route (LD_50_ = 375 µg/kg), followed by intranasal route (LD_50_ = 2000 µg/kg) and gastric intubation (LD_50_ = >5000 µg/kg). When ATX-a was administered together with MC-LR (31.3 µg/kg) by intranasal route, the LD_50_ decreased approximately 4-fold, at 500 µg/kg.	[29]
Crl:CD-1(ICR)BR mice	Single i.v. injection of 10–100 µg/kg (+) ATX-a or gavage doses of 0.098–15 mg/kg (+)ATX-a per day for 28 days Pregnant female mice were dosed by gavage 2.46 mg/kg (+)ATX-a daily between days 6–15 of pregnancy	Behavioral evaluation, assessment of locomotor activity and clinical observations	Animals showed salivation, hyperactivity and an increase in respiration after a single dose of toxin. The highest dose (100 µg/kg) produced the death in all treated mice. The NOAEL obtained in repeated doses was 0.098 mg/kg ATX-a. No adverse effects were observed in pregnant animals or their offspring. The NOAEL for teratogenicity was established at 2.46 mg/kg b.w.	[50]
Time-pregnant and non-pregnant CD-1 mice	I.p. dosages ATX-a ranged from 10 to 400 µg/kg in pregnant mice in a dose-finding assay Animals were treated with either 125 or 200 µg/kg for 5 days, on either GD 8–12 or GD 13–17 Mice received either 0, 500 or 1000 µg/kg of MC-LR by gavage and 50 min later, they received either 0, 500, 1000 or 2500 µg/kg ATX-a by gavage Mammalian embryos were exposed to 0.02, 0.2, 2.0 and 5.1 µg/mL ATX-a.	Evaluation for behavioral and physical alterations Analysis of morphogenesis by observation.	Adverse effects included difficult breathing, convulsions or altered gait in pregnant mice. At 200 µg/kg ATX-a, a reduced motor activity was observed and at the highest doses (300 and 400 µg/kg toxin), a 100% of mortality occurred. Nevertheless, no significant postnatal effects were observed in pups from any treatment group. No deaths were observed at any of the dose groups treated with MC-LR and ATX-a. Mammalian embryos exposed to 2.0 and 5.1 µg/mL showed perturbations in mouse yolk sac vasculature.	[73]
Female Sprague Dawley rats	Administration of 1, 2, 3.5 or 7 mM ATX-a by microdialysis probe (~281.3, 562.6, 984.55 or 1969.1 µg/mL) Toxin also was administered after exposure to different nicotinic or muscarinic receptors antagonists (MEC, MLA, atropine, α-bgt)	Determination of dopamine and metabolites by HPLC	An increase in striatal dopamine levels was produced in a dose-dependent way. There were not changes on release of dopamine metabolites. The combined used of ATX-a and different drugs indicated that ATX-a acts through nicotinic receptors. These results also support further *in vivo* evidence that α/β and α7 * nicotinic AChRs are involved in the striatal dopamine release induced by ATX-a.	[88]
Male Long Evans rats	Subcutaneous injections administered once a week for 4 weeks of 0.075–0.225 mg/kg (+)ATX-a or 0.20–0.95 mg/kg (±)ATX-a	Motor activity testing during 30 min sessions	Both forms, (+)ATX-a and (±)ATX-a, produced a reduction in locomotor activity horizontally and vertically after the first administration of the toxin. Weekly treatment did not change the effectiveness of the toxin. However, higher doses of racemic toxin were necessary to produce the acute effects. Neither form of toxin induced tolerance.	[89]
Male Long Evans rats	Four weekly subcutaneous injections of ATX-a doses ranged from 0.05 to 0.2 mg/kg	Behavioral study in trained rats	The toxin produced a dose-dependent reduction in response and reinforcement rates with the first administration. Tolerance was seen in behavioral responses after repeated administration with most doses, except for the highest dose (0.2 mg/kg ATX-a).	[90]
Male mice	Daily administration of 50, 100 or 150 µg/kg ATX-a by i.p. injection for seven days	Sperm counts and histopathological examinations on the testes	Dose-dependent reductions in epididymis weights and sperm count in all treatment groups. In addition, histopathological changes were observed, such as loosening of germ cells or degenerations in seminiferous tubules.	[91]
Female Sprague Dawley rats	3.5 mM ATX-a (~984.55 µg/mL) was administered by microdialysis probe into the striatum Toxin also was administered together with MLA	Measurement of amino acids content by HPLC	Toxin increased levels of extracellular glutamate, GABA, taurine and dopamine. The combined used of ATX-a and MLA indicated that glutamate release depended on the activation of α7 nicotinic receptors.	[92]
Female Swiss albino mice	Doses of ATX-a by gavage, i.p. injection or feeding	Determination of LD_50_ using OECD 425 guideline	Mice were more sensitive to i.p. injection exposure. LD_50_ obtained was 0.231 mg/kg for i.p. injection, 10.6 mg/kg for gavage and 25 mg/kg for feeding.	[93]

AchE: acetylcholinesterase; AchR: acetylcholine receptor; AP: alkaline phosphatase; α-bgt: α-bungarotoxin; b.w.: body weight; CE: choline esterase; ECAP: evoked compound action potentials; ED: effective dose; EROD: ethoxyresorufin-O-deethylase; GABA; gamma aminobutyric acid; GD: gestation day; GGTP: gamma glutamyl transpeptidase; GPT: glutamic pyruvic transaminase; GST: glutathione S-transferase; HPLC: high-performance liquid chromatography; i.p.: intraperitoneal; i.v.: intravenous; LC: lethal concentration; LC–MS/MS: liquid chromatography–mass spectrometry; LD: Lethal dose; LDH: Lactate dehydrogenase; MEC: mecamylamine; MLA: methyllycaconitine; MLD: Minimum lethal dose; NAR: nicotine-acetylcholine receptors; NOAEL: no observed adverse effect level; OECD: Organization for Economic Co-operation and Development; qPCR: quantitative polymerase chain reaction; UHPLC: Ultra high-performance liquid chromatography.

### 3.1. Aquatic Organisms

Several studies showed toxic effects in aquatic organisms by ATX-a, such as crustaceans [70,71,78,80], embryos or fertilized eggs of various fish or amphibian species [72,73,75] and fish [30,68,69,74,76,79].

The studies on the crustacean *Artemia salina* were intended to evaluate the ability of this organism to detect cyanobacterial toxicity [70,71]. The first study showed that the pure toxin was not toxic to *Artemia* larvae, whereas in both works, *Anabaena* strains produced abnormal swimming and increased the death percentage (LC_50_ = 2–14 mg/L) of *Artemia*. Similarly, *Daphnia* is a common test organism used in toxicological experiments due to its high sensitivity, and the ATX-a effects on this organism have also been reported [77,78,80]. Pure ATX-a was reduced to 33% survivorship of *D. pulex* after the exposure to 1.66 mg/L ATX-a for 24 h [77]. Bownik and Pawlik-Skowronska [78] determined other sensitive and early parameters of this organism exposed to ATX-a, such as behavioral and physiological responses. Changes in swimming speed, heart rate, thoracic limb activity and oxygen consumption were observed in a concentration- and time-dependent manner. These findings suggest that the analyzed parameters may be considered early indicators of ATX-a toxicity. More recently, Schwarzenberger and Martin-Creuzburg [80] went one step further and investigated the effects of ATX-a on life history parameters and gene expression of nicotine-acetylcholine receptors (NAR) of *D. magna.* The treatment with ATX-a produced by a strain of *Tychonema bourrelyi* caused a reduction in growth rates of *D. magna*, as well as an up-regulation of NAR gene expression. In addition, the rise of NAR gene expression was transferred maternally to the offspring, which means higher fitness of the descendant.

On the other hand, the effects of ATX-a were evaluated at different life stages of fish. Oberemm et al. [72] exposed embryos from zebrafish by immersion in solutions that contained pure ATX-a or crude extracts of *Anabaena flos-aquae* NRC-44-1. The only effect reported was a temporary alteration in heart rate at the highest concentration of pure toxin assayed (400 µg/L). The extract of cyanobacteria did not show any effects on the development of zebrafish. Moreover, Rogers et al. [73] observed a concentration-dependent transient narcosis, edema, loss of balance and 100% mortality in toad embryos exposed to 0.03, 0.3, 3.0 or 30 mg/L ATX-a for 10 days. Osswald et al. [74] also reported the effects of ATX-a on early stages of fish development. Fertilized eggs from *Cyprinus carpio* were incubated with pure ATX-a and cyanobacterial extracts containing ATX-a at higher concentrations than those tested by Oberemm et al. [72]. Pure neurotoxin had no effect on mortality at any concentration tested, whereas the cyanobacterial extract produced an increase in mortality as a function of time and concentration, reaching 100% mortality at the highest concentration used (666 µg/L ATX-a) after 4 days of exposure. These authors also analyzed skeletal malformations with similar results; significant differences were obtained only with cell extracts. Moreover, Osswald et al. [74] determined the toxicological effects of juvenile fish immersion in solutions with different concentration of *Anabaena* containing ATX-a. This treatment exposure produced abnormal swimming in all concentrations and the highest concentration assayed (10^7^ cells/mL) was 100% lethal after 24 h of treatment. The bioaccumulation of ATX-a by common carps also was determined, and levels of ATX-a ranged between 0.031 and 0.768 µg/g d.w. after exposure to 10^5^ and 10^7^ cells/mL, respectively [74]. This fact could indicate a possible transference of ATX-a to the higher levels of the food chain, and consequently, as mentioned in the introduction to this work, it could generate a risk to the health of consumers. However, contradictory results have been obtained. Thus, recently, Colas et al. [79] reported that ATX-a did not bioaccumulate in fish tissues (muscle, liver and gut) after 3 days using an oral; there was no observed adverse effect level (NOAEL) dose of 6.67 µg/g. More bioaccumulation studies are needed for this area.

Furthermore, different routes of exposure to ATX-a have been investigated on adult fish. It has been observed that the amount necessary to produce death in fish is different depending on the exposure route: Osswald et al. [30] reported a LD_50_ of 0.36 µg/g when the administration was i.p., while the levels necessary to reach the LD_50_ by gavage were more than 30 times higher (LD_50_ = 11.5 µg/g) [79]. Moreover, it should be noted that the neurotoxicological effects were similar in different species of fish exposed to ATX-a, specifically carp [74], zebrafish [76] and medaka fish [79]. These findings suggest a similar mechanism of action. In addition, the administration by i.p. injection of sublethal ATX-a doses ranging from 0.08 to 0.31 µg/g produced alterations in some hepatic and muscle enzyme activities such as GST, EROD, AChE and lactate dehydrogenase (LDH) [30].

### 3.2. Birds

Despite the reported incidence of ATX-a poisoning in wild birds such as flamingos [10,11,94] and ducks [17,95,96], there are very few studies conducted in these species. It should also be noted that the existing ones are very old, and no studies have been carried out with pure ATX-a in birds. Thus, only two studies have shown the adverse effects produced by *Anabaena flos-aquae* containing ATX-a in birds [68,69]. These authors reported different sensitivity to ATX-a in two avian species when administration was oral: pheasants required 2.4 times the dose of cell suspensions to reach the LD_90_ compared to ducks.

### 3.3. Mammals

Early research with mammals reported the clinical effects produced by exposure to *Anabaena flos-aquae* NRC-44, a cyanobacteria producer of ATX-a [68,69,81,82]. Tremors, altered gait, paralysis of respiratory muscles and even death by respiratory failure are characteristic symptoms of acute toxicity of ATX-a [69,81,82]. It is worth mentioning that all of the studies with mammals have been carried out with rodents, with the exception of Carmichael et al. [82], who considered adverse effects in calves, showing a similar response following treatment with *Anabaena flos-aquae* NRC-44-1.

Similar to *in vitro* models, a different toxicity has been identified depending on the stereoisomers of ATX-a [85,86,89]. A study compared the LD_50_ of single i.p. administration of (+)ATX-a, (−)ATX-a and racemic anatoxin-a in mice. The results showed that (+)ATX-a is the more potent enantiomer (LD_50_ = 386 µg/kg), followed by racemic isoform (LD_50_ = 913 µg/kg) and (−)ATX-a enantiomer, which showed a minimal effect [85]. Another study confirmed this difference, and LD_50_ of 85 and 400 µg/kg for (+)ATX-a and (±)ATX-a, respectively, were obtained after exposure by intravenous (i.v.) injection in rats [86].

Moreover, various administration routes of ATX-a have been investigated, with i.p. injection as the most studied route [28,29,73,82,91,93]. Results of administration by i.v. injection showed that it was the most effective route of administration (LD_50_ = 85 µg/kg bw in rat) [86]. The i.p. administration LD_50_ in mice was 260–315 µg/kg bw [28,85], followed by the intranasal route (LD_50_ = 2000 µg/kg bw) and, finally, oral administration, which required higher doses (LD_50_ > 5000 µg/kg bw) [28,29,93]. These results indicate a different kinetic behavior depending on the route of administration, with i.p. injection being more than 10 times more toxic than the oral one. Thus, they show complementary rather than comparable results. Thus, these results highlight the fact that toxicity studies based solely on i.p. injection might not provide a good estimate of the risk for human health since it does not represent a real route of the exposure that occurs in nature to this toxin.

So far, only two works considered the effects of repeated exposure for a long period of time to the neurotoxin ATX-a [50,83]. Thus, in the first study 2 groups of 20 female Sprague Dawley rats were exposed to 0.51 or 5.1 mg/kg ATX-a in drinking water for 54 days and no adverse effects were detected after the treatment. Astrachan et al. [83] also determined the effect of repeated doses of 0.016 mg toxin by i.p. injections for 21 days without changes in the studied parameters. The results suggested that ATX-a did not produce significant effects when the concentrations are lower than those causing acute effects. Moreover, a 28-day study was carried out in mice with 0.098, 0.49 or 2.46 mg/kg of (+)-ATX-a by gavage and the NOAEL obtained was 98 µg/kg [50]. For this reason, WHO considered the available toxicological information as not adequate to develop a long-term health-based reference value for ATX-a, with special emphasis on the need to carry out more studies in this regard [16].

Some authors have investigated the effects on locomotor activity of this neurotoxin exposure in rodents. Stolerman et al. [87] and MacPhail et al. [89] detected a reduction in locomotor activity after exposure to both forms of (+)ATX-a and racemic form. Moreover, the second author administered sublethal doses of toxin weekly for 4 weeks without developing tolerance to the toxin. In contrast, another study showed tolerance to ATX-a in the behavior when weekly doses were administered to trained rats [90]. In all these experiments ATX-a was compared with nicotine and both showed similar—but not identical—behavior, suggesting that the sites of action in the nervous system may be different [87,89,90].

The information on the effects of ATX-a on development and reproduction is limited. Pregnant hamsters received three i.p. injections of 0.125 or 0.2 mg/kg bw of ATX-a in different stages of gestation. This treatment did not produce any malformations, but stunting was observed in 10–20% of fetuses compared with controls [83]. A developmental toxicity study on pregnant mice administered with 2.46 mg/kg (+)ATX-a daily for 5 days by gavage was carried out by Fawell et al. [50]. Any adverse effects were noted in either pregnant animals or the fetus, so the NOAEL for teratogenicity at 28 days was established at 2.46 mg ATX-a/kg/per day [50]. In another study, Rogers et al. [73] observed significant alterations such as disturbances in the yolk sac vasculature of mouse embryos. Regarding the effect of the toxin to the male reproductive system, a study with repeated doses of ATX-a (50, 100 and 150 µg/kg per day) for 7 days in male mice was carried out and showed a significant reduction in sperm count as well as other adverse effects in the testes such as a loosening of germ cells or degeneration in seminiferous tubules [91].

### 3.4. Plants

Few studies have shown the adverse effects of this neurotoxin in plants and all of them have focused mainly on the analysis of oxidative stress parameters [97,98,99,100,101] (see Table 3).

The first report employed the aquatic plant Lemma minor, and the macroalga *Chladophora fracta* exposed to concentration of ATX-a ranged from 0.1 to 25 µg/mL. The highest concentrations triggered an increase in enzymatic activities related with oxidative stress such as CAT, POD and GST accompanied with a reduction in photosynthetic oxygen production [97]. Oxidative damage was also detected in the only study in terrestrial plants after 5 µg/L ATX-a exposure. Thus, the activity of antioxidant enzymes, such as SOD or GR, was elevated with this treatment. Moreover, an inhibition of root growth was observed in alfalfa seeds [98]. At lower concentrations, ranging from 0.5 to 50 µg/L, the activation of antioxidative systems was also observed in the aquatic plant *Ceratophyllum demersum* [99]. When the exposure concentration was 15 µg/L ATX-a, a maximum enzyme activity was produced at 24 h of treatment; after this treatment time, the antioxidant enzyme activities began to decrease until almost recovering control levels at 336 h of exposure. These authors also analyzed the effects in growth parameters of a sub-chronic exposure to ATX-a for eight weeks in *C. demersum*, resulting in an inhibition of fresh weight gain that was observed following one-week ATX-a exposure [100]. Li et al. [101] detected a plant defense response against ATX-a in *Vallisneria natans*, which produced phytohormones such as abscisic acid and strigolactone. Likewise, a significant influence on biofilms was detected in the presence of ATX-a [101]. These findings demonstrated that both aquatic and terrestrial plants (after irrigation with contaminated water events) may suffer adverse effects due to the presence of ATX-a.

## 4. Conclusions

In conclusion, toxicological studies of ATX-a to date are very scarce in comparison to other cyanotoxins. The need for more *in vitro* and *in vivo* studies following OECD guidelines should be emphasized, mainly in standardized neuronal cell lines and *in vivo* studies under conditions that simulate what can occur in nature (longer periods of exposure, oral route, etc.) for risk assessment purposes. Toxicological aspects of great interest such as mutagenicity, genotoxicity, immunotoxicity and alteration of hormonal balance especially need to be studied in depth. Of particular interest is the bioaccumulation capacity of this toxin in animals and plants, as contradictory results have been reported. The elucidation of the accumulative potential of ATX-a is essential for regulating the limits of this toxin in water and food to guarantee the health of consumers and to prevent possible intoxications.

## 5. Material and Methods

### 5.1. The Information Sources and Search Strategy

The search for information was performed through the electronic research databases Web of Science, Scopus, Science Database and PubMed until September 2022. The following keywords were selected to be used in all search engines: anatoxin-a, cyanotoxins, *in vitro*, *in vivo*, toxicity, genotoxicity, mutagenicity, cytotoxicity. In addition, the bibliography of these articles has been reviewed to complete the search.

### 5.2. Eligibility and Exclusion Criteria

The following criteria were taken into account in the information selection process:

Inclusion criteria: (1) articles on ATX-a toxicity *in vitro*; (2) articles on ATX-a toxicity *in vivo*; (3) articles published prior to September 2022; (4) articles reporting comprehensive results published in internationally recognized journals.

Exclusion criteria: (1) articles on ATX-a toxicity in field studies; (2) articles published in a language other than English; (3) proceedings of conferences and dissertations; (4) abstracts only available.

In relation to the risk of bias due to the quality of the studies considered, the majority of studies have a low (61%) and medium (31%) risk of bias compared to a minority (7%) with a high risk of bias. In general, the studies with a high risk of bias are those that are older in date of publication (see Table S1 in Supplementary Material).

Moreover, all of the articles contained in Table 1, Table 2 and Table 3 have been taken into account for the elaboration of Figure 2, Figure 3, Figure 4 and Figure 5.

## Figures and Tables

**Table 1:** *In vitro* toxicity studies carried out with ATX-a in different experimental models.

Experimental Models	Experimental Conditions	Assays Performed	Main Results	References
Isolated muscle preparations of *Rana pipiens* frog-rectus abdominus, chick biventer cervicis, rat phrenic nerve hemidiaphragm, cat sciatic nerve-anterior tibialis and guinea pig ileum	0.5 to 10 µM of (~0.0826–1.65 µg/mL) extracted or synthetic ATX-a.	Muscle response/maximal response.	Extracted and synthetic ATX-a had similar pharmacological properties. ATX-a had a potency greater than acetylcholine or carbachol on striated muscle. Tetrodotoxin had no significant effect on ATX-a responses. ATX-a had ganglionic stimulating effects on the smooth muscle of guinea pig ileum. ATX-a showed less potent but qualitatively similar action to decamethonium.	[43]
Electric organs of *Torpedo ocellata* and neural membranes of male Wistar rats	Log (−8 to −5.5) M of ATX-a for 5 min (~0.0017–0.522 µg/mL)	Binding assays: Measurement of the radioactivity associated with the tissue trapped on the filters	ATX-a stimulated the “ion channel blockers” such as [^3^H]perhydrohistrionicotoxin, [^3^H]phencyclidine and [^3^H]phencyclidine methiodide with a range of EC_50_ from 0.14 to 0.28 µM for these effects. EC_50_ of ATX-a for inhibition of 3-[^3^H]quinuclidinyl benzilate binding was between 10 and 20 µM, and a low affinity of this toxin for the muscarinic acetylcholine receptors of rat brain was shown.	[44]
Isolated muscle preparations of frog-rectus abdominus and hindfoot of the *Rana pipiens* Electric organs of *Torpedo californica*	Log (−8 to −4) M (~0.0017–16.52 µg/mL) of (+)ATX-a or (−)ATX-a 10 µM (~1.65 µg/mL) of (+)ATX-a for 5 min for binding measure	Potency assay. Binding assays: Measurement of the radioactivity associated with the tissue trapped on the filters Electrophysiological techniques such as patch clamping	(+)ATX-a was a more potent agonist than ACh or carbamylcholine because of a higher affinity for the nicotinic acetylcholine receptor, whereas (−)ATX-a was less potent than carbamylcholine. At various concentrations the toxin activates the appearance of channels with the same conductances as ACh-induced channels but with a shorter lifetime.	[45]
Cortical brain rat tissue	Log (−10 to −4) M (~0.000017 to 16.52 µ/mL) of (+)ATX-a, (−)ATX-a or (±)ATX-a	[^3^H]ACh binding assay: measurement of the radioactivity associated with the tissue trapped on the filters.	(+)ATX-a (IC_50_ 4.5 nM) was 160-fold more potent than the (−)ATX-a (IC_50_ 750 nM) and 20-fold more potent than (±)ATX-a (IC_50_ 30 nM) in inhibiting [^3^H]Ach binding: (+)ATX-a > (±)ATX-a > (−)ATX-a Hill coefficients of 0.56, 0.44 and 0.52 were obtained for (+)ATX-a, (±)ATX-a and (−)nicotine, respectively.	[46]
Rat hippocampal synaptosomes Mouse M10 cells (that expressed chicken α4β2 nAChR subunits) *Xenopus* oocytes (α7 nAChR) Fetal rat hippocampal neurons	Log (−9 to −3) M (~0.00017 to 165.237 µg/mL) of (+)ATX-a	Patch-clamp technique was used to record whole-cell currents from fetal rat hippocampal neurones cultured for 20–45 days Activation of the nAChR was measured as the stimulation of ^86^Rb+ influx into the cells by treatment with the agonist for 1 min Conventional dual-electrode voltage clamp for electrophysiological recording	The EC_50_ of (+)ATX-a in presynaptic nAChR, α4β2 nAChR, α7 nAChR and hippocampal neurones were 1.4 × 10^−7^, 4.8 × 10^−8^, 5.8 × 10^−7^ and 3.9 × 10^−6^, respectively. This toxin was between 3–50 times more potent than (−)-nicotine and 20 times more potent than acetylcholine. (+)ATX-a is the most efficacious nicotinic agonist.	[47]
Bovine adrenal chromaffin cells	ATX-a: 0.1–100 µM (~0.0165–16.52 µg/mL) for 5 min.	Release of catecholamines: High pressure liquid chromatography for catecholamines separation and detection	ATX-a was a potent agonist of the neuronal-type nicotinic receptor. It evoked higher secretion of noradrenaline and adrenaline than nicotine (EC_50_ 1–2 µM vs. EC_50_ 4–5 µM). Mecamylamine (at 10 µM for 5 min) inhibited the catecholamines secretion produced by ATX-a (at 5 µM for 5 min). At high concentrations of ATX-a (10 µM) the release of noradrenaline and adrenaline in the presence of 50 mM additional K^+^ was similar to that of ATX-a alone.	[48]
Synaptosomes of male Sprague Dawley rats	0.01–100 µM ATX-a (~0.00165–16.52 µg/mL) by superfusion	Measurement of radioactivity each two minutes in a Packard scintillation spectrometer	[^3^H]dopamine was released in striatal synaptosomes in a concentration-dependent way after treatment with the toxin. The EC_50_ was 0.11 µM. This release was dependent of Ca^2+^.	[49]
*In vitro* organs bath preparations: Guinea-pig ileum Rat phrenic nerve diaphragm Chick biventer cervicis *In vivo* experiments: see Table 2	ATX-a: 0.005–0.5 µg/mL	% response of tissue	ATX-a was 7-, 136- and 24-fold more potent as an agonist than nicotine in guinea-pig ileum, rat phrenic nerve diaphragm and chick biventer cervicis, respectively. The addition of the hexamethonium, a ganglion blocker, produced a parallel shift in the dose response curves for ATX-a and nicotine. Authors suggested a guideline value for ATX-a in drinking water of 1 µg/L.	[50]
Rat brain slices	Log (−10 to −3) M (~0.000017 to 165.237 µg/mL) of (+)ATX-a for 5 min	Concentration–response assays of nicotinic agonist-evoked release of [^3^H]-norepinephrine	ATX-a produced the concentration-dependent release of [^3^H]-NE in slices of hippocampus, thalamus and cortex. Concentration–response curve-revealed values of EC_50_ = 0.23, 0.13 and 0.15 µM in the hippocampus, thalamus and frontal cortex, respectively. Compared with other agonists, the rank order of potency was (±)-epibatidine >> (+)ATX-a > A85380 > DMPP = NIC = (−)-cytisine.	[51]
Rat thymocytes and African green monkey kidney cells (Vero).	ATX-containing cell free extracts from *Anabena flos aquae* (ACE) (10 to 50 µg/mL) or purified (+)ATX-a (1–10 µg/mL) for 15 min, 3 h, 6 h or 24 h of treatment depending on the assay	Cytotoxicity was measured by trypan blue exclusion assay, LDH leakage and MTT test Apoptosis by fluorescence staining and TUNEL assay Agarose gel electrophoresis for DNA fragmentation analysis Fluorescence for ROS quantification Fluorimetric assay for determination of caspase activity	ATX-a produced cytotoxicity, apoptosis and caspase-3 activation in both cell types. ACE and ATX-a induced ROS generation in rat thymocytes in a concentration- and time-dependent manner. ATX-induced apoptosis was mediated by caspase activation and ROS generation.	[52]
Spleen cells isolated from male BALB/c mice	0.1 µg/mL ATX-a for 4, 24 or 48 h.	Cytotoxicity by MTT assay Apoptosis by flow cytometry	Time-dependent decrease in cell viability. Cytotoxic effects in a non-selective and non-specific manner. Both lymphocyte subpopulations (T and B cells) showed to be in late apoptotic or secondary necrotic phases after ATX-a exposure for 4 h.	[53]
*Salmonella typhimurium* TA 1535/pSK1002	Toxin concentrations (ATX-a and ATX-a + MC-LR) were 0.25, 0.5, 1 and 2 μg/mL for 2 h.	umuC Easy CS Genotoxicity Assay kit to determinate the growth factor and β-galactosidase activity by spectrophotometry	In absence of S9 fraction, genotoxic effects and an increase in β-galactosidase activity at 0.5–2 µg/mL and 0.25–2 µg/mL ranges were observed for ATX-a and ATX-a + MC-LR mixture, respectively. In the presence of S9 fraction, no effects were detected in any samples. No effects in the growth factor in presence and absence of S9 fraction.	[54]
Lymphocytes of common carp	0.01, 0.1, 1, 5 and 10 µg/mL of ATX-a for 24 h	Cytotoxicity by CellTiter-Glo^®^ Luminescent Viability assay Determination of cell death type by cellular DNA fragmentation ELISA test kit Caspase-Glo^TM^ 3/7 Assay MTT Test for lymphocyte proliferative activity	A slight decrease in ATP levels and mild necrosis was observed only at the highest concentration tested. Cell apoptosis was observed after 24 h of exposure to 1, 5 and 10 µg/mL of ATX-a. Moreover, an early stage of apoptosis in these cells was confirmed by increased activity of effector caspases 3/7. The toxin also decreased the proliferation ability of lymphocytes in a concentration-dependent manner.	[55]
Immune cells from common carp	0.01–1 µg/mL of ATX-a for 24 h	Cytotoxicity by bioluminescent assay, GSH assay and ROS production assay	Decreased ATP levels were not observed. ATX-a produced an increase in ROS at 0.01 and 0.025 µg/mL and a reduction in the respiratory burst activity at the highest concentration (1 µg/mL) in pronephros phagocytes. In blood phagocytes, the increase in ROS was observed at 0.05 µg/mL.	[56]
Head kidney leukocytes and blood leukocytes from common carp	Toxin concentrations (ATX-a and ATX-a extract) were 0.01 or 0.1 µg/mL for 4 h.	Gene expression of IL-1β, TNF-α, IL-10 and TGF-β cytokines by RT-PCR	Pure ATX-a dysregulated the expression of pro-inflammatory cytokines IL-1β and TNF-α more promptly than the anti-inflammatory cytokines TGF-β and IL-10. In general, pure ATX-a produced a significant increase in IL-1β, TNF-α and IL-10 expression in both cellular models. However, at 0.1 µg/mL, this toxin generated a significant decrease in TNF-α level. Contrary effects were observed after ATX-a extracts exposure in these cellular models. Thus, ATX-a extract produced a significant decrease in IL-1β and TNF-α expression levels. In addition, a significant increase in IL-10 level was observed after 0.1 µg/mL exposure in both cellular models. TGF-β was increased only in head kidney cells exposure to range of 0.01–0.1 µg/mL.	[57]
*S. typhimurium* TA98, TA100, TA1535, TA1537 and *Escherichia coli* WP2 uvrA and WP2 [pKM101]	Pure ATX-a in a range of 0.312–10 µg/mL. Mixture of ATX-a, CYN and MC-LR at 1 µg/mL. Different cyanobacterial extracts containing ATX-a, CYN and/or MC-LR.	Ames Test	Pure ATX-a and its mixture with CYN and MC-LR did not show mutagenic or cytotoxic effects. Some extracts containing cyanotoxins showed mutagenic effects in TA98 and TA100 bacterial strains. The results indicated that while tested cyanotoxins were not directly responsible for the observed mutagenicity of the extracts analysed, some synergistic interactions with other unidentified cyanobacterial-derived factors involved in the process were possible.	[58]
Common carp leukocytes	0.5 µg/mL of ATX-a for 18 h	Comet assay	No genotoxic effects were observed in cells exposed to ATX-a.	[59]
Murine macrophage-like RAW264.7, microglial BV-2 and neuroblastoma N2a cell lines	0.1 and 10 µM (~0.0281 and 2.813 µg/mL) of ATX-a Equimolar mixture of ATX-a, CYN and MC-LR at 0.001, 0.1 and 10 µM.(~0.000281, 0.0281 and 2.813 µg/mL)	Cellular viability by the MTT assay Apoptosis by measurement of the activation of caspases 3/7 Measurement of TNF-α protein by ELISA test	ATX-a did not reach LD_50_ levels of toxicity in any cell type, whereas equimolar mixture of toxins reached LD_50_ between 0.1–10 µM at different times (24, 48 or 72 h). The toxin mixture induced cell death of the N2a cells in a dose- and time-dependent manner. A measurement of 10 µM of toxins mixture induced almost a total death of RAW264.7 and BV-2 cells. Cytotoxicity: ATX-a < equimolar mixture of toxins in N2a cells < BV-2 cells < RAW264.7 cells ATX-a produced an induction of caspase activity, mainly when it is contained in the mixture of toxins. Moreover, N2a cells showed a higher sensitivity to ATX-a (alone or in the mixture) as compared to RAW264.7 and BV-2 cells. ATX-a significantly increased TNF-α secretion only in N2a cells.	[60]
Genetically modified strains of *Saccharomyces cerevisiae* (yeast cells)	7.1 × 10^−11^ to 9.1 × 10^−5^ M (~0.00002 to 25.6 µg/mL) of ATX-a for 24 h	Flow cytometry for cell viability. YES assay for estrogenic response detection Q-Exactive Tandem Mass Spectrometry for detection the intermediate products of ATX-a	A significant reduction in viability in yeast cells was only observed after 4.5 × 10^−5^ M ATX-a exposure. ATX-a simulates endocrine-disrupting chemicals as it modulates the 17β-estradiol-induced estrogenic activity, resulting in non-monotonic dose responses. After the treatment with a high activity catalyst system (Fe^III^-B */H_2_O_2_), the ATX-a degradation products presented insignificant changes in its estrogenic activity. ATX-a was shown to induce estrogenic activity as agonist in the YES assay.	[61]
*Microcystis* spp., *Anabaena variabilis* and *Selenastrum capricornutum*	25 µg/L of ATX-a 25 µg/L of ATX-a combined with 25 µg/L of MC-LR for 4 days	Flow cytometry to count cell density Fluorescence using Turner Designs TD-700 fluorometer to quantify chlorophyll-a concentration in the cultures ELISA kits for toxin quantification and measurement of antioxidant enzyme activities (SOD, POD, GST) GC for measurement of N_2_ fixation rates	ATX-a (alone or in mixture with MC-LR) produced a significant decrease in cell density and chlorophyll-a levels in *Microcystis* sp, and produced the opposite effects in *S. capricornutum*. In *Anabaena*, no changes were observed in these parameters after 4 days of exposure to the toxins. ATX-a increased antioxidant enzyme activities in *Microcystis* sp, which were unchanged or decreased in *Anabaena* UTEX B377 and *S. capricornutum*, respectively. ATX-a significantly inhibited nitrogen fixation by *Anabaena* UTEX B377. In general, the combined effects of these cyanotoxins were often more intense than their individual effects on some strains.	[62]
Lymphocytes of *Carassius auratus*	0.01–10 mg/L of ATX-during 12 h	Analysis by electron microscopy, flow cytometry, electrophoresis and assay kits for antioxidant parameters	Vacuolation, swollen mitochondria and DNA fragmentations induced by ATX-a. Apoptosis in a concentration-dependent manner. Oxidative stress (↑ ROS and MDA; ↓ SOD, CAT, GR, GPx and GSH).	[63]
Human keratinocytes	0.1, 1 or 10 µg/mL of ATX-a for 24, 48 or 72 h	WST-1 cell proliferation and crystal violet assay for proliferation Cytotoxicity detection kit (LDH) Scratch assay to describe the migratory activity of human keratinocytes	Cristal violet assay: in the proliferation of human keratinocytes, a toxic effect on the cells was only observed under the influence of the highest studied concentration. WST-1 assay: Toxic effects at 1 and 10 µg/mL. At 10 µg/mL, the decrease in cell proliferation was 60%, 81% and 84% after 24, 48 and 72 h, respectively. LDH: The toxicity of ATX-a was 24% after long incubation (48 h) at 10 µg/mL. No influence on keratinocyte migration was observed.	[64]

ACE: Anatoxin Containing Extract; ACh: Acetylcholine; ADN: deoxyribonucleic acid; ATP: adenosine triphosphate; CAT: Catalase; DMPP: 1,1-dimethyl-4-phenylpiperazine; EC_50_: Mean effective concentration; ELISA: enzyme-linked immunosorbent assay; Fe^III^-B */H_2_O_2:_ catalytic oxidation system; GC: Gras chromatography; GPX: Glutathione peroxidase; GR: Glutathione reductase; GSH: Glutathione; GST: Glutathione S-transferase; IL: interleukin; LDH: Lactate dehydrogenase; MDA: Malonaldehyde; MTT: methyl thiazole tetrazolium; nAChR: nicotinic acetylcholine receptor; NE: Norepinephrine; NIC: Nicotine; PCR: polymerase chain reaction; POD: Peroxidase; ROS: Reactive oxygen species; RT-PCR: Real-time polymerase chain reaction; SOD: Superoxide dismutase; TGF-β: transforming growth factor beta; TNF-α: tumor necrosis factor Alpha; YES: yeast estrogen screen.

**Table 3:** Studies on plants exposed to ATX-a.

Experimental Model	Experimental Conditions	Assays Performed	Main Results	References
*Lemma minor* and *Chladophora fracta*	5–25 µg/mL ATX-a for 4 days 0.1–20 µg/mL of ATX-a for 7 days for *L. minor*	Measurement of POD, CAT and GST activities and protein content Measurement of the macrophyte photosynthetic oxygen production	An increase in POD activity was observed in both organisms with the highest toxin concentration (25 µg/mL) after 4 days of treatment. Exposure of 7 days produced a rise in CAT and GST activities in *L. minor* at 5 and 20 µg/mL ATX-a. Moreover, these concentrations of ATX-a decreased oxygen production.	[97]
Alfalfa (*Medicago sativa*)	5 µg/L ATX-a for 7 days	Morphological changes Oxidative stress parameters	Toxin produced a 27-fold inhibition on development of primary root of alfalfa compared to the control. Similarly, oxidative stress was produced. An increase in LPO and SOD, POD and GR activities was observed, as well as a decrease in CAT and GST activities.	[98]
*Ceratophyllum demersum*	0.005–50 µg/L ATX-a for 24 h or 14 days	Oxidative stress parameters Analysis of chlorophyll and carotenoid contents by spectrometry	Concentrations greater than 0.5 µg/L led an inhibition of fresh weight. Toxin also decreased chlorophyl *a* content at 5 and 50 µg/L. H_2_O_2_ levels and GST, POD, SOD, GR, MDAR and APX activities were increased in a concentration-dependent manner.	[99]
*Ceratophyllum demersum*	15 µg/L (±)ATX-a for 8 weeks	Oxidative stress parameters Analysis of chlorophyll contents by spectrometry Determination of growth parameters	The toxin produced oxidative stress. An increase in H_2_O_2_ levels and GST, POD, SOD, GR and APX activities were observed. Moreover, changes in chlorophyll contents were produced. Inhibition of fresh weight gain detected after 1 week exposure.	[100]
*Vallisneria natans*	0.05–5 µg/L ATX-a or 0.05–5 µg/L MC-LR + ATX-a	Measurement of enzymatic biomarkers Determination of phytohormones by ELISA Analysis of biofilms	The toxin induced changes in oxidative stress biomarkers. An increase in CAT, POD and SOD activities and GSH content were observed. ATX-a also produced a rise in phytohormones and altered biofilms. A decrease in the biomass of plants was produced in all groups treated. Combined toxin treatment produced a reduction in SOD and POD activities compared with single toxin, showing an antagonistic effect.	[101]

APX: ascorbate peroxidase; CAT: catalase; ELISA: enzyme-linked immunosorbent assay; GR: glutathione reductase; GSH: glutathione; GST: glutathione S-transferases; H_2_O_2_: hydrogen peroxide; LPO: lipid peroxidation; MDAR: monode-hydroascorbate reductase; POD: peroxidase; SOD: superoxide dismutase.

## Data Availability

Not applicable.

## Supplementary Materials

The following supporting information can be downloaded at: https://www.mdpi.com/article/10.3390/toxins14120861/s1, Table S1: Risk of bias for the methodological quality of studies reporting the toxic effects produced by ATX-a under laboratory conditions. 0: not reported; 1: not appropriately or clearly evaluated; 2: appropriately evaluated. M: medium (4–6); L: low (7–8); H: high (0–3).
