# Supplementary material for: Toxic Effects Produced by Anatoxin-a under Laboratory Conditions: A Review
Source: Toxins (Basel). 2022 Dec 8;14(12):861. doi: 10.3390/toxins14120861 (PMC9784168; doi:10.3390/toxins14120861)
Supplement: Supplementary file 1 [file toxins-14-00861-s001.zip › toxins-2034948-supplementary.pdf]

toxins

MDPI

Supplementary Materials: Toxic Effects Produced by Anatoxin-a Under Laboratory Conditions: A Review

Cristina Plata-Calzado, Ana I. Prieto, Ana M. Cameán and Ángeles Jos

Table S1. Risk of bias for the methodological quality of studies reporting the toxic effects produced by ATX--a under laboratory conditions. 0: not reported; 1: not appropriately or clearly evaluated; 2: appropriately evaluated. M: medium (4-6); L: low (7-8); H: high (0-3).

Reference	Clear objective	Well characterized product	Reproducibility of the assay	Adequate statistical analysis	Total	Risk of Bias
[4]	2	2	1	0	5	M
[28]	2	2	1	2	7	L
[29]	2	2	1	0	5	M
[30]	2	2	2	2	8	L
[43]	2	2	1	0	5	M
[44]	2	1	1	0	4	M
[45]	2	1	1	0	4	M
[46]	2	1	1	0	4	M
[47]	1	1	1	0	3	H
[48]	2	2	2	0	6	M
[49]	2	2	1	2	7	L
[50]	2	2	1	0	5	M
[51]	2	2	1	2	7	L
[52]	2	2	2	2	8	L
[53]	2	2	2	2	8	L
[54]	2	2	2	2	8	L
[55]	2	2	2	2	8	L
[56]	2	2	2	2	8	L
[57]	2	2	2	2	8	L
[58]	2	2	2	2	8	L
[59]	2	2	2	2	8	L
[60]	2	2	2	2	8	L
[61]	2	2	2	2	8	L
[62]	2	2	2	2	8	L
[63]	2	2	2	2	8	L
[64]	2	2	2	2	8	L
[68]	1	1	1	0	3	H
[69]	1	1	1	0	3	H
[70]	2	2	1	0	5	M
[71]	2	1	1	0	4	M
[72]	1	2	1	2	6	M
[73]	2	2	2	2	8	L
[74]	2	2	1	0	5	M
[75]	2	2	2	2	8	L
[76]	2	2	2	0	6	M
[77]	2	2	2	2	8	L
[78]	2	2	2	2	8	L
[79]	2	2	2	2	8	L

[80]	2	2	2	2	8	L
[81]	1	1	1	0	3	H
[82]	2	1	1	0	4	M
[83]	2	1	1	2	6	M
[84]	2	1	1	2	6	M
[85]	2	2	1	0	5	M
[86]	2	2	1	2	8	L
[87]	2	2	1	2	7	L
[88]	2	2	2	2	8	L
[89]	2	2	2	2	8	L
[90]	2	2	2	2	8	L
[91]	2	2	2	2	8	L
[92]	2	2	2	2	8	L
[93]	2	2	2	2	8	L
[97]	2	1	1	2	6	M
[98]	2	2	2	2	8	L
[99]	2	2	2	2	8	L
[100]	2	2	2	2	8	L
[101]	2	2	2	2	8	L
